# Data linkage and computerised algorithmic coding to enhance individual clinical care for Aboriginal people living with chronic hepatitis B in the Northern Territory of Australia – Is it feasible?

**DOI:** 10.1371/journal.pone.0232207

**Published:** 2020-04-28

**Authors:** Kelly Hosking, Geoffrey Stewart, Mikaela Mobsby, Steven Skov, Yuejen Zhao, Jiunn-Yih Su, Steven Tong, Peter Nihill, Joshua Davis, Christine Connors, Jane Davies

**Affiliations:** 1 Primary Health Care Branch, Top End Health Service, Northern Territory Government, Darwin, Northern Territory, Australia; 2 Global and Tropical Health Division, Menzies School of Health Research, Darwin, Northern Territory, Australia; 3 Centre for Disease Control, Northern Territory Government, Darwin, Northern Territory, Australia; 4 Innovation & Research, Northern Territory Government, Darwin, Northern Territory, Australia; 5 Victorian Infectious Disease Service, Royal Melbourne Hospital, Melbourne, Victoria, Australia; 6 Peter Doherty Institute for Infection and Immunity, Melbourne, Victoria, Australia; 7 Department of Infectious Diseases, John Hunter Hospital, Newcastle, New South Wales, Australia; Centre de Recherche en Cancerologie de Lyon, FRANCE

## Abstract

**Background:**

Chronic hepatitis B (CHB) is endemic in the Aboriginal population of Australia’s Northern Territory (NT). However, many people’s hepatitis B virus (HBV) status remains unknown.

**Objective:**

1. To maximise the utility of existing HBV test and vaccination data in the NT by creating a linked dataset and computerised algorithmic coding. 2. To undertake rigorous quality assurance processes to establish feasibility of using the linked dataset and computerised algorithmic coding for individual care for people living with CHB.

**Methods:**

Step 1: We used deterministic data linkage to merge information from three separate patient databases. HBV testing and vaccination data from 2008–2016 was linked and extracted for 19,314 people from 21 remote Aboriginal communities in the Top End of the NT. Step 2: A computerised algorithm was developed to allocate one of ten HBV codes to each individual. Step 3: A quality assurance process was undertaken by a clinician, using standardised processes, manually reviewing all three databases, for a subset of 5,293 Aboriginal people from five communities to check the accuracy of each allocated code.

**Results:**

The process of data linking individuals was highly accurate at 99.9%. The quality assurance process detected an overall error rate of 17.7% on the HBV code generated by the computerised algorithm. Errors occurred in source documentation, primarily from the historical upload of paper-based records to electronic health records. An overall HBV prevalence of 2.6% in five communities was found, which included ten cases of CHB who were previously unaware of infection and not engaged in care.

**Conclusions:**

Data linkage of individuals was highly accurate. Data quality issues and poor sensitivity in the codes produced by the computerised algorithm were uncovered in the quality assurance process. By systematically, manually reviewing all available data we were able to allocate a HBV status to 91% of the study population.

## Introduction

Chronic hepatitis B (CHB) infection is a serious public health challenge, with an estimated 292 million people living with CHB infection worldwide, and a global prevalence estimated at 3.9% [1]. CHB disproportionately affects Indigenous populations globally [2] and this is similar in Australia. A recent systematic review and meta-analysis showed an overall prevalence of 10.8% in pre and 3.5% in post-universal vaccination populations in Aboriginal and Torres Strait Islander people (hereafter respectfully referred to as Aboriginal) in Australia [3]. CHB is endemic in Aboriginal people of the Northern Territory (NT) of Australia, with a contemporary estimated prevalence of 6.1% [4]. However, surveillance and epidemiological data are often absent [5].

In 1988, the NT was one of the first places in the world to introduce a universal newborn and infant hepatitis B virus (HBV) vaccination program for Aboriginal children, which expanded to include all children in the NT in 1990 [6, 7]. A catch-up vaccination program was introduced in 1998 for children aged 6–16 years old. A study of a cohort of antenatal women, using NT notification data and the midwifery dataset, showed a decrease in HBV prevalence in Aboriginal women born since the introduction of HBV vaccination in comparison to those born in the pre-vaccine era (2.2% versus 3.5%) but found HBV prevalence remained substantially higher for Aboriginal women compared with non-Indigenous women (2.4% versus 0.04%) [6, 7].

It is estimated that without appropriate management and treatment 25% (15–40%) of people living with CHB will die from liver disease [8, 9], namely liver failure or liver cancer. CHB is the main risk factor for developing liver cancer [10]. NT Aboriginal people have a sub-genotype of HBV—C4, which has only been identified in this population [11]. C4 has genotypic markers associated with faster progression to cirrhosis and liver cancer [12] and NT Aboriginal people have been shown to have six times the incidence of liver cancer compared to non-Indigenous people [13]. Liver disease is the third most significant contributor to the gap in life expectancy between Aboriginal and non-Indigenous Australians [14]. These adverse outcomes can be prevented with available, publicly funded treatments [9, 15, 16].

A foundation step to improving health outcomes and preventing deaths is to identify all people living with CHB. With the aim of reducing the burden of CHB, Australia’s National Hepatitis B Strategy highlights Aboriginal people as a priority population [17, 18] and sets clear targets to improve the cascade of care: 80% of the population to be diagnosed and aware of their infection; 50% of people living with CHB engaged in care; and 20% of people living with CHB to be on treatment [18]. In response to this National Strategy, a key component of the NT Hepatitis B Action Plan developed in 2014 is to determine the HBV status of all Aboriginal people [19]. In this setting, many people’s HBV status remains unknown [4, 5].

To determine the HBV status of an individual, which can be infected, immune or non-immune, HBV testing and review of HBV vaccination data (including date and time of administration) is required. Hepatitis B virus testing requires a venous blood sample for the following three tests: hepatitis B surface antigen (HBsAg), hepatitis B core antibody (Anti-HBc), hepatitis B surface antibody (Anti-HBs). Current serological testing platforms available in Australia for HBV are highly specific and sensitive [20]. However, difficulties can arise as in addition to having all three serological tests processed and results available, the results need to be appropriately interpreted and actioned by a skilled clinician.

With almost 60,000 Aboriginal people living in the NT [21], the potential to determine each individual’s HBV status with an automated process offers obvious advantages over a manual audit. Additionally, a highly mobile population [22] and serological testing using different pathology providers [4] means that individual health services may not have access to all available data for an individual, with negative implications for accurate HBV status determination. Data linkage is an important method to better utilise existing but separate datasets for surveillance, epidemiology, administrative and public health purposes [23–26] and in supporting clinical care on a general level [27]. Data linkage for direct individual care is less well defined [28]. Data linkage was used successfully in Victoria, Australia, to improve completeness of Aboriginal status reporting for communicable disease notifications, including HBV [26].

Using a devised computerised algorithm we aimed to link each individual’s HBV test and vaccination data from three electronic patient databases, in order to piece together all relevant clinical information pertaining to their HBV status. The HBV code produced correlated to a clinical HBV status description, which could then be applied to the individual’s primary care electronic health record (EHR) and used to assist in appropriate clinical care. Before allocating the HBV code generated by the algorithm to an individual record, a thorough manual review quality assurance process was undertaken on a subset of the linked dataset to assess if it was fit for this purpose. The findings of this quality assurance process will add to the commentary of whether data linkage can be used for direct clinical care.

## Methods and materials

The Northern Territory Hepatitis B Action Plan [19] in 2014 led to a broad multi-sectorial health service agreement on the objective of determining the hepatitis B virus (HBV) status of all Aboriginal people in the NT. In line with research ethics and guidelines [29–31], we conducted extensive community consultation with Aboriginal Health Boards, Health Service Managers and health centre staff to obtain permission to commence and undertake the study. The study design and methodology was borne directly from the community consultation process, which included requests to use all available pathology data before requesting further blood tests. The project relies on identifiable data obtained from primary care and public health data sources, and involves the input of data back into the primary care clinical information systems in NT services. The dataset analysed was not anonymous, it had the individual’s hospital record number (HRN) available so that the clinician reviewing the data could ensure that each individual was connected to an appropriate care pathway. This was discussed with community and Aboriginal Health Boards. Collection of identifiable data has ethics approval. Ethical approval was granted through the Human Research Ethics Committee of the Northern Territory Department of Health and Menzies School of Health Research–HREC 2015–2417.

For definitions of the terminology used for this study, refer to Table 1.

**Table 1:** Definitions of the terms used for this study.

Term	Definition
Data linkage	The process of linking an individual (and their data) from three separate electronic data systems, using deterministic linkage, with the individual’s hospital record number (HRN) as the unique identifier
Linked dataset	The dataset containing the linked individual’s details from the data linkage process, including demographic data and all available HBV tests and vaccination data
HBV code	The code (number) allocated to each individual based on a combination of HBV test and vaccination data as assigned by the computerised algorithm. This code number appears on the linked dataset for each individual
HBV status	The determined and allocated HBV status (which can be immune, infected, non-immune, unknown) based on HBV serologic markers +/- vaccinations (if born before 1990) or vaccination +/- serological marker (prioritising serological markers) if born since 1990 and received primary vaccine course as an infant and with no identifiable HBV transmission risk factors
Manual review	The (quality assurance) process of a trained clinician manually reviewing all available HBV test and vaccination data for each individual from three data systems to determine and allocate a HBV status.

### Step 1: Deterministic data linkage of three separate patient databases

**Fig 1:**
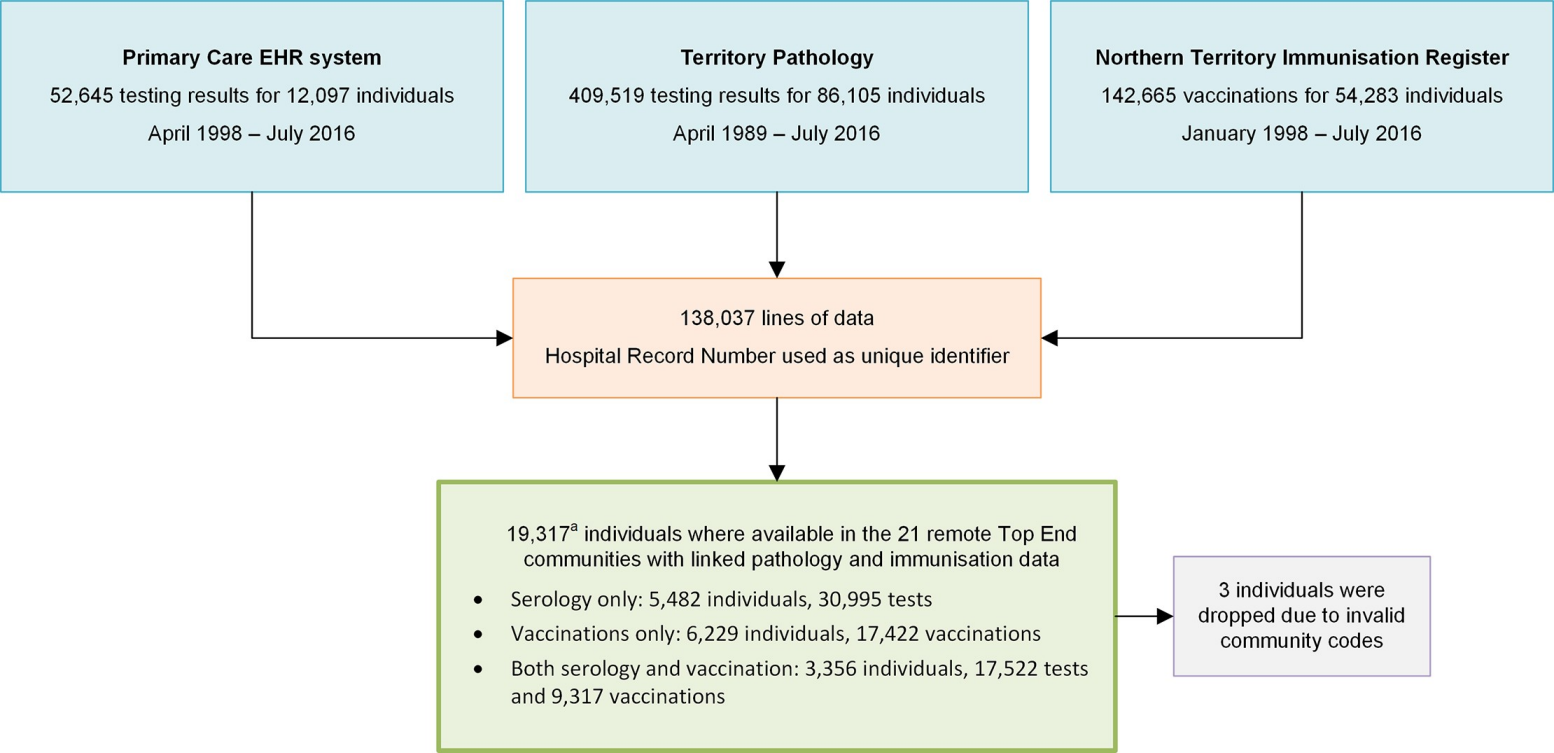
Flow diagram detailing sources of pathology and vaccination data and production of dataset, with duplicate tests and individuals removed. ^a^includes non-Indigenous residents of the remote communities.

All individuals with a record in the primary health EHR system from 21 remote Aboriginal communities in the Top End of the NT were included in the data linkage study. Each individual who has ever accessed public health services in the NT has a unique individual hospital record number (HRN). The HRN is based on the NT Department of Health’s Client Master Index that records demographic data, Indigenous status and residency status. The Client Master Index is a highly accurate source of NT population demographic data [32]. We used deterministic linkage using the HRN as a unique identifier to link HBV testing (462,164 tests) and vaccination (142,665 vaccinations) data from three patient databases for 19,314 individuals, (Fig 1). The linkage and data extraction took place in July 2016.

The patient databases reviewed were:

1. Primary care EHR system: One of the two primary care EHR systems used within remote primary health care (PHC) centres in the NT was used. All residents of the community have a primary health record on this system, into which client demographic and health data are entered and can be extracted. From the primary care EHR system we extracted demographic data and HBV serological markers from January 2008 to July 2016 and vaccination data from 2000.
2. NT Immunisation Register (NTIR): This is an all of life immunisation register (adult vaccinations available since 2016) managed by the NT Centre for Disease Control. The primary care EHR service centre provides a weekly report to the NTIR staff, who manually enter and upload vaccinations from all vaccine providers and patient databases in the NT. This vaccine data is then uploaded automatically from NTIR to the Australian Immunisation Register (AIR) twice weekly. HBV vaccination data since 1991 was extracted from this system.
3. Territory pathology: This is part of the patient database used by all of the public hospitals in the NT. An individual has a record in this system if they have ever visited a NT public hospital. HBV serological markers since 2008 were extracted from this system.

HBV serological markers from January 2008 to July 2016 were extracted based on searchable health-level 7 (HL7) coding which applies an individual code to each serological marker. Extraction of HBV serological markers prior to 2008 was not possible from the primary care EHR as these earlier historic results were not allocated searchable HL7 codes. As such, these serological markers could not be extracted for inclusion for linkage but were visible within the EHR system and were considered when determining a HBV status as part of the manual review quality assurance process.

Duplicate records were removed (notably vaccinations recorded in both primary health EHR and NTIR and repeat episodes of HBV testing over time) to produce a single linked dataset, which included the most recent HBV test result available (see Fig 1).

### Step 2: A computerised algorithm was developed and used to allocate one of ten HBV codes to each individual

**Fig 2:**
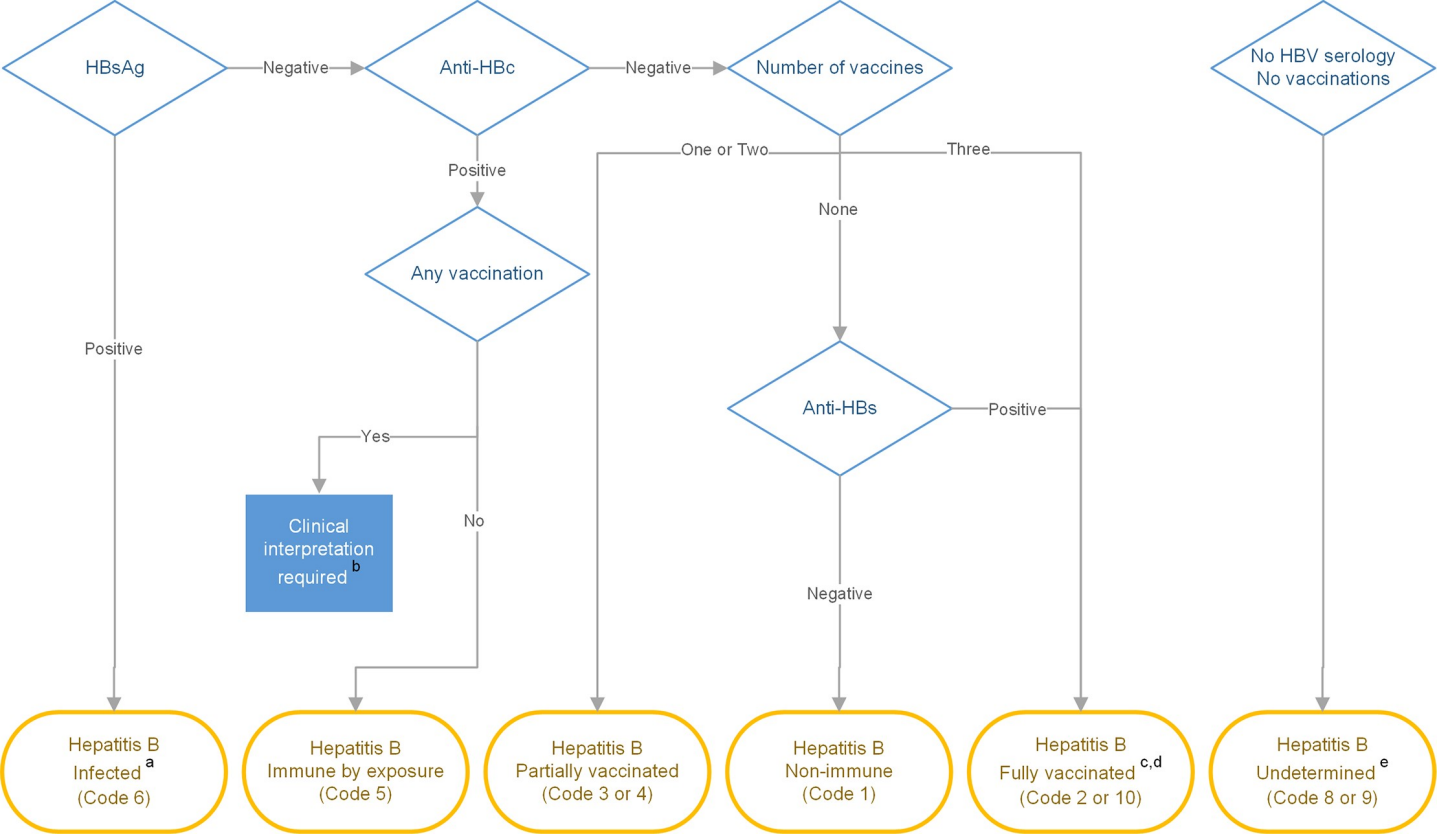
Algorithm used to allocate a code number to each individual. ^a^ If HBsAg positive apply regardless of other data. ^b^ Anti-HBc positive in the presence of any vaccines resulted in interrogation of clinical record to determine the temporal relationship between the HBV test and administration of vaccines. ^c^ All people born since 1990 with 3 documented vaccination, given at correct intervals, were coded as fully vaccinated, even if HBV tests were not available. ^d^ Isolated Anti-HBs positive in the absence of either Anti-HBc or any record of vaccination were allocated a HBV code of presumed fully vaccinated. ^e^ Recall added to record for HBV test, no HBV status added to primary care EHR.

An algorithm developed for the study determined allocation to the HBV codes, as described in Table 2 and Fig 2. Ten codes were developed to identify the clinical requirements of each person to facilitate accurate recalls for clinical care. The algorithm is an AND not OR algorithm–i.e. a person needs to fulfil all of the four column criteria to be allocated to that code. Positive HBsAg indicates active infection, positive Anti-HBc is a marker of immunity from infection (past [if HBsAg negative] or current [if HBsAg positive]); and positive Anti-HBs indicates immunity (due to vaccination [if HBsAg negative and Anti-HBc negative] or due to resolved infection [if HBsAg negative and Anti-HBc positive]). We used STATA (Statacorp, College Station, Texas) version 13 to apply the algorithm to all individuals in the study cohort and then assign a HBV code number to each of them accordingly. See S1 Appendix, decision tree for the algorithm to automate and assist clinical decisions using vaccination and pathology data.

**Table 2:** HBV coding numbers and computerised algorithm for determining HBV status.

Code number	HBV status	Vaccinations	HBsAg^a^	Anti-HBc^b^	Anti-HBs^c^
**1**	Non-immune	None or missing	Negative	Negative	Negative
**2**	Fully Vaccinated	3 doses	Missing or negative	Missing or negative	Negative, positive or missing
**3**	Partially vaccinated, needs 1 dose	2 doses	Missing or negative	Missing or negative	Negative, positive or missing
**4**	Partially vaccinated, needs 2 doses	1 dose	Missing or negative	Missing or negative	Negative, positive or missing
**5**	Immune by exposure	Prioritise serological markers	Negative	**Positive**	Negative, positive or missing
**6**	Chronic infection	Prioritise serological markers	**Positive**	Negative, positive or missing	Negative, positive or missing
**7**	Clinical interpretation required^d^	Prioritise serological markers	Negative or Positive	**Positive** or Negative	Negative or positive
**8**	No data	None or missing	Missing	Missing	Missing
**9**	Insufficient data	None or missing	Missing or negative	Missing or negative	Missing or negative
**10**	Presumed fully immunised	None or missing	Negative	Negative	Positive (i.e. >10)

^a^ HBsAg–hepatitis B surface antigen, if positive indicates active infection

^b^ Anti-HBc–hepatitis B core antibody, if positive is a marker of immunity from infection (past or current)

^c^ Anti-HBsAb–hepatitis B surface antibody, if positive (>10IU/ml) indicates immunity (from vaccination or infection)

^d^ Individuals with discrepant results that need a clinician to review in order to interpret, including previous Anti-HBc positive but most recent test result Anti-HBc neg

Five consistent clinical descriptions of an individual’s HBV status were agreed upon to allow systematic documentation of HBV status in the primary care EHR systems. The HBV status descriptions align with local guidelines (28) and are: i) HepB: Fully vaccinated (relating to code numbers 2 and 10, Table 2); ii) HepB: Immune by exposure (relating to code number 5, Table 2); iii) HepB: Infected on treatment (relating to code 6, Table 2); iv) HepB: Infected NOT on treatment (relating to code 6, Table 2); v) HepB: Non-immune (related to code 1, Table 2).

### Step 3: Quality assurance process by clinician

The quality assurance process commenced in October 2016. We selected five communities (out of the 21) with a total population of 6,728 where the majority (78.7%) were Aboriginal people. These communities were selected as consultation had already been held and permission obtained. Due to the transient nature of the people living in these communities, population numbers fluctuate. However, in October 2016, at the beginning of this study the Aboriginal population range was 241 for the smallest community to 2,769 for the biggest community. The distance to the nearest hospital in Darwin, the capital of the NT, ranges from 250 km to 509 km. During the monsoonal season, two of the communities have no road access to Darwin for up to four months of the year. Two communities are islands that have no resident doctor and are only accessible on small aircraft or boat.

A trained study nurse undertook a manual review quality assurance exercise on the linked dataset to assess the accuracy of the coding process implemented through data linkage and the application of the computerised algorithm. This quality assurance process aimed to achieve a number of goals: to ensure true matches (i.e. the data linked is for the same individual); to assess if all available vaccination and pathology data had been linked and extracted and that HBV codes generated by the computerised algorithm were correct; to document the clinician determined HBV status on the primary care EHR; to ensure all individuals were allocated to appropriate care pathways; and to determine the HBV prevalence among the residents of the five communities.

**Fig 3:**
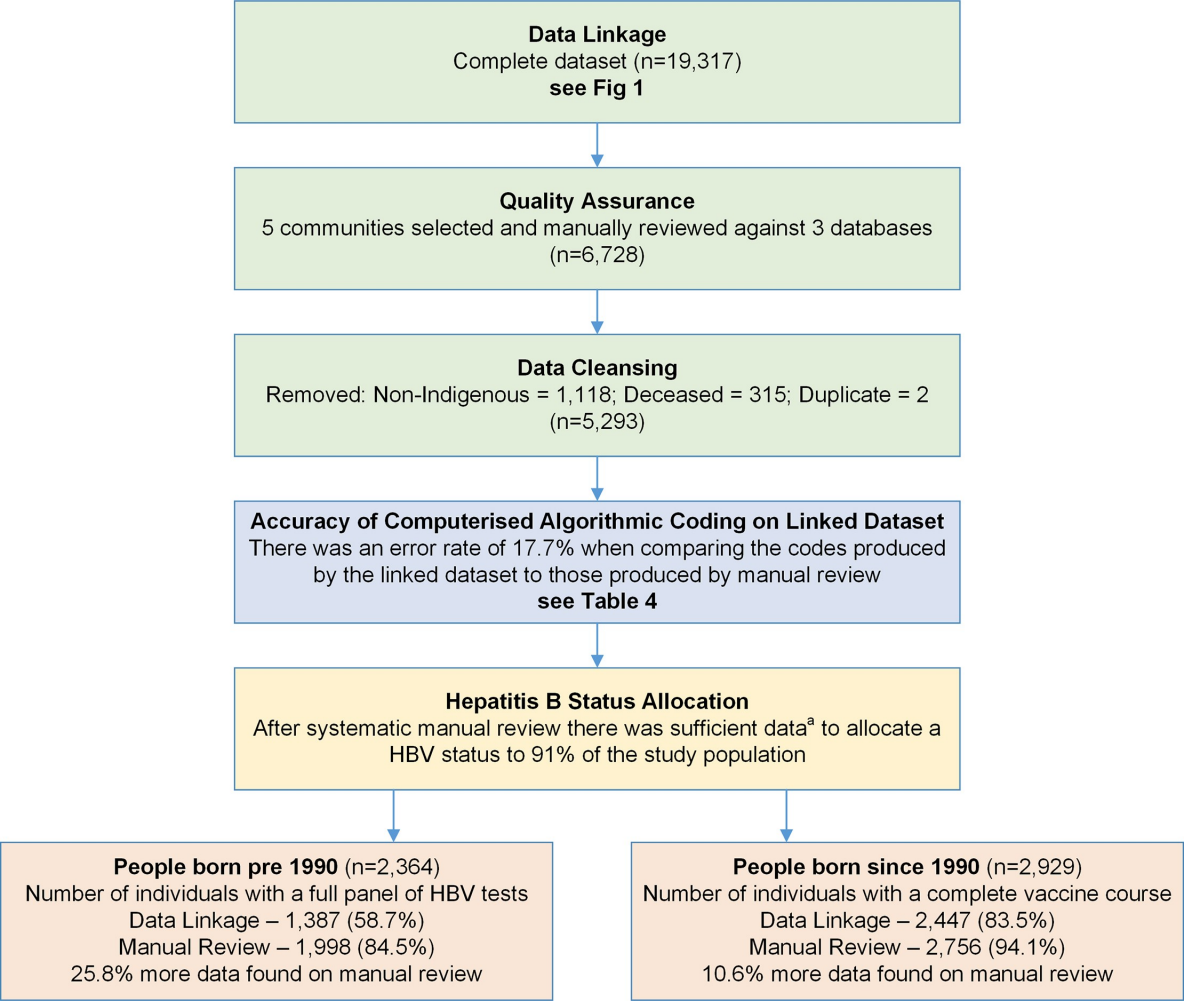
Quality assurance on code generated by computerised algorithm on linked dataset. ^a^ Suffient data is defined as full panel of HBV tests for people born before 1990 and all people born since 1990 is 3 documented vaccination, given at correct intervals (+/- HBV test data).

The study nurse checked the pathology and immunisation data for each individual from the three patient databases, determined the individual’s HBV status based on all available information and compared it against the code generated by the computerised algorithm. The HBV status was then documented on the individual’s primary care EHR record to ensure the individual was allocated to appropriate care pathways. Any necessary recalls were also added—i.e. if the individual did not have enough data to determine a HBV status, a recall for HBV testing was created. If an individual had a positive HBsAg (HBV infected) a “Hep B Infected” care plan was created and local health staff informed. The accuracy of each code generated by the computerised algorithm was compared against the manual review. The study nurse initially audited a random selection of 400 people (5.9% of the study population), checking the sample records against the original data from the three data sources. We detected an error rate of 16.7% from the data linking, which persisted despite attempts to refine the process. See S2 Appendix for details of the algorithm development process and iterations. The study nurse documented on the dataset spreadsheet whether the HBV code determined by the manual review matched that determined by the process of data linking and computerised algorithm. The linked dataset was cleaned at the end of the quality assurance process to remove duplicate patient records and records of deceased and non-Indigenous people (see Fig 3).

The accuracy of the data linkage of individuals was assessed using the concordance correlation coefficient, to measure reproducibility of shared information from different data sources [33]. The accuracy of the linked data and the subsequent HBV code generated for each individual based on the computerised algorithm’s interpretation of this data was evaluated by a clinician manually reviewing all available data. The accuracy was calculated by the number of individuals per code which on manual review had the same code as the computerised algorithm divided by the number of individuals the computerised algorithm assigned to that code x100. The 95% confidence intervals for prevalence estimates were calculated using the exact binomial method. All data was analysed using STATA (Statacorp, College Station, Texas) version 15.

## Results

### Results of the process of data linking individuals from three separate patient databases

The quality of the deterministic linkage using the hospital record number (HRN) as a unique identifier was highly reliable in linking the three selected datasets, with over 99.9% matching agreement measured by concordance correlation coefficient [33].

Of the total 19,314 individuals included in the study, 42.1% (n = 8,133) had a record of a HBV test, 49.6% (n = 9,585) had a record of a HBV vaccination and 75.8% (n = 14,631) had either a HBV test or a HBV vaccination (see Table 3).

**Table 3:** Number of HBV blood tests peformed and HBV vaccinations received (per individual), Top End, Northern Territory, 2016 shown on data linked dataset.

Blood tests^a^		Number of HBV vaccinations				Total
for HBV	1	2	3	> = 4	No record	
**1–2**	151	248	666	158	1696	2919
**3–4**	171	187	456	153	1740	2707
**5–6**	70	101	172	112	783	1238
**> = 7**	98	88	128	128	827	1269
**No record**	401	1447	3940	710	4,683	11181
**Total**	891	2071	5362	1261	9729	19314

^a^ HBV test means one or more of the panel of tests (could be HBsAg and/or Anti-HBc and/or Anti-HBs). A person tested for both HBsAg and Anti-HBs in the same testing panel would be counted as having one test.

Of those born since 1990 (when universal HBV vaccination was included in the NT childhood immunisation schedule) 22.7% had a hepatitis B test, 82.6% had a vaccination (one or more), 89.3% had a test or vaccination and 16% had both test and vaccination. For those born in the pre-vaccination era (before 1990), 60.2% had a hepatitis B test, 19.0% had a vaccination (one or more), 63.1% had a test or vaccination and 16% had both test and vaccination.

### Results of the quality assurance process

We compared the HBV codes produced by the data linking and computerised algorithmic process against those produced from a manual review, these results are presented in Table 4.

**Table 4:** HBV code allocated by data linking and computerised algorithim process for the Aboriginal population in five communities, compared to code determined by clinician’s manual review of the clinical information in three patient databases.

HBV code	Number of individuals predicated by the data linkage computerised algorithm	Number of individuals identified by manual review	Accuracy^a^ of data linkage coding (%)	Sensitivity	Specificity	PPV	NPV
**Code 1: Non-immune**	174	48	27.6%	40%	49%	28%	99%
**Code 2: Fully vaccinated**	2,903	2,852	98.2%	81%	50%	100%	76%
**Code 3 & 4: Needs 1 or 2 doses**	138	25	18.1%	37%	100%	17%	99%
**Code 5: Immune by exposure**	738	738	100%	82%	100%	100%	96%
**Code 6: Chronic infection**	119	119	100%	97%	100%	100%	99%
**Code 7: Discrepant results**	38	6	15.8%	100%	100%	15.8%	100%
**Code 8: No data**	366	216	59%	100%	100%	59%	100%
**Code 9: Insufficient data**	302	87	28.8%	92%	100%	29%	100%
**Code 10: Presumed immunised**	515	265	51.4%	96%	100%	67%	100%
**Total Correct rate**	**5,293**	**4,356**	**82.3%**				

^a^ Accuracy = trained clinician manually reviewed each individual per code and determined the number of individuals correctly allocated to each code i.e. Number of individuals per code which on manual review had same code as computerised algorithm/Number of individuals the computerised algorithm assigned to that code x100

Inaccuracies were not evenly distributed across the 10 codes. In two clinically significant codes, representing either past or current infection [20], the agreement rate was extremely high—100% for “Code 5: immune by exposure” and 100% for “Code 6: chronic infection”. However, four people (3.3% of all cases) with a positive HBsAg (HBV infected) were incorrectly assigned to a variety of other unrelated codes. Reviewing the individual cases, all had an available HBsAg positive result since 2008 on one or more EHR systems and we were unable to identify any process issues that lead to the error.

Of note, 162 people who were immune by exposure were incorrectly allocated through the data linkage and computerised algorithmic coding process to a random variety of other codes. As this group has the potential for reactivation of HBV in the presence of immunosuppression [9, 20], emphasis was placed on correctly coding all individuals. Overall, of the 5,293 individuals from the selected Aboriginal population, the HBV code was a correct match for 4,356, an accuracy of 82.3% with incorrect coding occurring in 17.7% (937 out of 5,293).

### Descriptive assessment of gaps in the data extraction

Of the 5,293 Aboriginal people living in the five study communities 2,368 were born before 1990. The data linkage and computerised algorithmic coding extracted HBV tests on 1,387 (58.6%) individuals in this cohort but on manual review 1,998 individuals (84.4%) had a HBV test, meaning that 25.8% of available HBV testing data was not extractable. The main source of error occurred due to HBV tests prior to 2008 which were not entered into the EHR as HL7 coded data, and so could not be extracted as part of the data linkage and computerised algorithmic coding process, but were visible when the EHR was reviewed manually.

For vaccination data on individuals born since 1990, of the 2,929 individuals in this cohort, the data linkage and computerised algorithmic coding extracted (a completed course of) HBV vaccination data for 2,443 (83.4%) compared to 2,756 individuals (94%) who could be allocated to this HBV status “fully vaccinated” from manual review, demonstrating that 10.7% additional complete vaccination data was identified through the manual review process (see Fig 3). There were multiple error sources observed, primarily related to transcription errors at the time of introduction of the primary care EHR system and vaccination documentation errors meaning they were unable to be extracted.

### CHB prevalence in the five communities assessed in the quality assurance process

We identified 123 people with CHB in this study from the 5,293 individuals manually audited. Excluding those with no or insufficient data to determine a HBV status (n = 489), the population prevalence was 2.6% (95% CI 2.1–3.0%). The CHB prevalence in the pre-vaccination cohort (born before 1990) was 5.2% and the prevalence in people born after 1990 was 0.2%. See Table 5 for age (at time of audit, July 2016) and sex distributions among people living with CHB. Of people born since 1990, 600 out of 2,929 (20.5%) had a full HBV test, of which four (0.7%) were HBsAg positive (HBV infected). Of those living with CHB, 9% were on antiviral treatment, this is a cross-sectional estimate of people who were on treatment at the time of the study and does not include the deceased or people who are no longer on treatment.

**Table 5:** People living with chronic hepatitis B, by age group and sex, in five remote communities in the NT.

	Female				Male				Total				
Age group^a^	Cases	Population	No data	Prevalence^b^	Cases	Population	No data	Prevalence	Cases	Population	No data	Prevalence	95% CI
**0–9**	0	463	19	**0.0%**	0	552	15	**0.0%**	0	1,015	34	**0.0%**	**0.0–0.4%**
**10–19**	0	527	8	**0.0%**	2	584	24	**0.4%**	2	1,111	32	**0.2%**	**0.02–0.7%**
**20–29**	3	563	31	**0.6%**	5	536	33	**1.0%**	8	1,099	64	**0.8%**	**0.3–1.5%**
**30–39**	18	412	68	**5.2%**	22	379	70	**7.1%**	40	791	138	**6.1%**	**4.4–8.2%**
**40–49**	13	349	54	**4.4%**	13	356	63	**4.4%**	26	705	117	**4.4%**	**2.9–6.4%**
**50–59**	8	180	38	**5.6%**	15	185	28	**9.6%**	23	365	66	**7.7%**	**4.9–11.3%**
**60–69**	4	83	19	**6.3%**	11	61	12	**22.4%**	15	144	31	**13.3%**	**7.6–21.0%**
**70–79**	1	25	3	**4.5%**	7	30	3	**25.9%**	8	55	6	**16.3%**	**7.3–29.7%**
**80–89**	1	4	0	**25.0%**	0	2	1	**0.0%**	1	6	1	**20.0%**	**0.5–71.6%**
**90–99**	0	1	0	**0.0%**	0	1	0	**0.0%**	0	2	0	**0.0%**	**0.0–84.2%**
**Sum:**	**48**	**2,607**	**240**	**2.0%**	**75**	**2,686**	**249**	**3.1%**	**123**	**5,293**	**489**	**2.6%**	**2.1–3.0%**

^a^ Age is the individual’s age at the time of study, July 2016

^b^ Prevalence is calculated as cases/(population-no data)*100

We found 10 individuals with CHB (8.1% of the total number of people living with CHB in the five communities) who were not identified as being infected on any system or EHR database. These cases had a positive HBsAg result available in one or more patient databases but were not engaged in care and had no initial assessment, counselling, follow up or care plan in place. As a result of the study findings, appropriate care plans have now been added to these primary care EHR systems in consultation with the health centre clinicians in the community.

There was sufficient existing HBV test and/or vaccination data to allocate a HBV status to 91% of the study population. Of which: 84% were immune–either through vaccination (n = 3,565) or past, resolved infection (n = 888); 214 (4%) people were HBV non-immune, meaning a HBsAg, Anti-HBc and Anti-HBs are all negative (and with no evidence of vaccination)–a hepatitis B vaccination care plan was added for these people; 9% (n = 489) of the population had insufficient data to determine a HBV status–HBV testing recalls were added to the primary care EHR so the individual could be offered an HBV test and appropriate action taken when results available.

## Discussion

Our study demonstrated an extremely high accuracy of using the deterministic data linkage method to link individuals from three clinical databases. In this study’s setting, of remote-dwelling Top End people in the NT (n = 19,315) there was a 99.9% matching rate using this data linkage method, measured by concordance correlation coefficient [33]. However, the quality assurance process uncovered a high error rate in the HBV codes produced by the computerised algorithmic coding system. These results provide evidence that implementing the algorithmic coding system is not feasible in this study context. From the systematic, manual review of all available HBV test and vaccination data from three data systems for each individual (some data of which was not extractable through the data linkage process) we were able to determine the HBV status of 91% of the study population and found an overall CHB prevalence of 2.6%.

The linked dataset results showed there were a number of individuals identified with missing variables and many demonstrating a level of over-testing, see Table 3. Once a HBV status of immune is established there is no need to test for HBV again unless the individual’s clinical situation changes, i.e. the individual becomes immunosuppressed or has abnormal liver function tests [9, 20]. This over-testing can be avoided by clear systems and processes and correct documentation of an individual’s HBV status, which would result in savings from unnecessary additional pathology and vaccinations. Strong, clear and sustainable systems are essential in the NT, which has high staff turnover and a heavy reliance on short-term agency staff [22, 34].

### Quality assurance process on a subset of linked data

There are two main types of linkage algorithms used in clinical research–deterministic and probabilistic [35]. Deterministic data linkage is sometimes called exact matching because it involves linking datasets based on a unique identifier [36]. Probabilistic data linkage uses statistical models and algorithms to estimate matches [35]. We evaluated the feasibility of data linkage for this research and concluded we could use deterministic linkage as it was possible to link datasets using the hospital record number (HRN) as the unique identifier.

We used a manual review process, which is a highly accurate tool for assessing data linkage quality [37, 38]. Our manual review quality assurance process on a subset of the population (6,728 out of 19,317) determined the accuracy of the data linkage and computerised algorithmic coding so we could assess the best way to achieve the highest accuracy in a cost-effective manner. As we wanted to utilise the data linkage and computerised algorithmic coding to assign individuals to clinical care pathways, with an emphasis on ensuring that all people living with CHB were correctly identified and engaged in care, we had a low threshold for error.

Evaluating linkage quality and impact of linkage error are important. Thresholds for balancing data linkage errors (type I and II) can be adjusted depending on the nature of the study [35]. As this research is practical, applied and aims to connect all CHB infected clients to care, we have taken a conservative approach. We aimed for a sensitivity of >99% for CHB cases i.e. true positives need to be identified as such. We also require a high sensitivity, of >95%, in the immune by exposure (Anti-HBc positive) HBV code. This is due to the clinical significance of these HBV statuses [20] and the potential negative implication of a mismatch. For instance, if a person with a positive HBsAg (living with CHB) is allocated to an incorrect and benign code they will not receive the monitoring and care they require and are therefore at risk of potential negative health consequences, such as CHB-related cirrhosis and liver cancer. All people who are Anti-HBc positive (but HBsAg negative) need to be correctly allocated to the “immune by exposure” HBV code as this group has the potential for reactivation of HBV in the presence of immunosuppression [20].

In this study, we have demonstrated that an automated computerised algorithmic coding system has the potential be used to determine individual’s HBV status, but that incompleteness of serological markers and vaccination data for inclusion in the linkage process resulted in an unacceptably high error rate for some HBV codes. Additional factors such as the timing of vaccinations and transcription errors at the time of introduction of the primary care EHR system also contributed to this error rate. As a result of the quality assurance process several refinements to the computerised algorithm were made, see S2 Appendix for more details on the development process and iterations. Several limitations in the computerised algorithm have been identified throughout the various iterations of both algorithm and clinical manual review audit process. In line with contemporary NT hepatitis B public health and vaccination guidelines the algorithm allocated 3 HBV containing vaccinations to the “fully vaccinated” code. We identified that such an approach failed to consider appropriate dose intervals for vaccination and that 62 individuals received vaccinations at intervals that were less that the minimum dosing intervals and were thus invalid. Additionally, the algorithm failed to distinguish individuals vaccinated according to the birth and infancy vaccination schedule and children and adults vaccinated later in life. These indiviuals require HBV testing to ensure they have not become HBV infected during the period in which they were susceptible to HBV, prior to vaccination. Given the natural history of CHB, that acquisition in infancy and childhood is more likely to lead to chronical infection [39], there would potentially be CHB infections in this cohort. In the manual review process this potential was considered (and adjusted for when calculating the error rate per code).

On an initial random selection of 400 individuals we detected an error rate of 16.7%, which persisted despite attempts to refine the process. Once all 5,923 individual’s records were manually reviewed we evaluated the sensitivity and specificity of the results of our data linkage and computerised algorithmic coding system and established that the overall error rate of 17.7% was too high to be used for informing direct individual patient care. Due to the clinical significance of the immune by exposure and infected HBV codes, the sensitivity of 83% and 97% respectively in these HBV codes was felt to be unacceptably low. As the overall goal of our study was to find all true cases of CHB and identify all of those immune by exposure in the NT, we focused on sensitivity for these two codes and assigned increased relative importance to the sensitivity over the positive predicative value.

Errors were detected in completely extracting all HBV test and vaccination data, which undermined the utility of the process to allocating an individual to the correct care pathway. The data linkage process could only extract HBV testing results since 2008. However, testing conducted prior to this was frequently visible in the EHR databases and included on recent results, which meant we were able to include results prior to 2008 when manual review of the records was performed, thereby increasing the number of people with available data.

We noted one source of error occurred in source documentation, predominantly from the historical upload of paper-based records to EHRs and with data in free text sections (e.g. vaccination information) not being extracted. This highlights problems with the quality of data organisation and documentation in each system. Similar findings have been found in other studies [40].

### Existing data and CHB prevalence

In the NT, there is currently a gap in documenting individuals’ HBV status. Although a contemporary study reported that 54% of Aboriginal people living in the NT have had HBV testing [4], a major gap in our public health response to HBV is establishing in a methodical way who is infected, who is immune and who has never been tested [18].

By using a clinician to review multiple data sources, we were able to obtain a more complete picture of an individual’s HBV status than could be ascertained by using a single data source. We also located HBV test and vaccination data recorded for individuals that the linking process did not detect. By systematically reviewing pathology and vaccination data across various systems, we could determine a HBV status, using existing data, for 91% of individuals in the five selected communities. This then supported our hypothesis that there would be sufficient existing information within the patient databases to be able to determine a HBV status of the majority of the study population.

These findings emphasised that existing clinical data is available but not always utilised for appropriate diagnosis and care pathway allocation as we would expect it to be. For people born before 1990 the data linkage and computerised algorithmic coding found 1,387 individuals out of 2,364 (58.7%) had full HBV testing. On manual review of these same individuals we found that 1,998 (84.5%) had HBV testing and could be allocated a HBV status, demonstrating that the manual review process increased data visibility and utility by 25.8% (see Fig 3). The amount of sufficient, existing HBV test and vaccination data available was higher than that reported in other studies [4].

Overall, the prevalence of CHB in this Aboriginal cohort of 5,293 was found to be 2.6%. In the pre-vaccination cohort (born before 1990) prevalence was 5.2%, dropping to 0.2% in the post vaccine cohort. These findings are consistent with other Australian data documenting a reduction in prevalence of HBsAg positivity when comparing pre and post vaccination eras [3, 4, 6]. There are also more males (61% of CHB infected) than females (39%) living with CHB in these communities. This finding of higher prevalence in males is consistent with other studies [3, 4]. Of those living with CHB, 9% were on treatment which is higher than estimates for the NT of 5.2% [41], however falls below the national target of 20% on treatment [18]. We expect that by employing a systematic process as used in this study, which included altering primary health care records, informing clinicians of all infections in their community and the addition of CHB care plans to the primary care EHR, treatment uptake will improve. This will be measured in follow up studies.

### Limitations of the study

Although we were able to gather data for a very high proportion of the population, we did not have 100% coverage. However, given that the age and sex distribution for the 9% with missing data is similar as that for the 91% with data we believe our results are representative of the entire study population. In-line with current NT public health guidelines [7], people born since 1990 who received 3 documented HBV vaccinations as an infant were coded as fully vaccinated—even in the absence of serological evidence that they are immune or not infected [7]. The exception to this is individuals with an identifiable risk factor for HBV transmission, i.e. born to a HBsAg positive mother or a household or sexual contact. These individuals require HBV testing before HBV status can be determined. Ideally, everyone should have a HBV test, to allow complete and accurate data on an individual level, ensuring that all people living with CHB are diagnosed and aware of their infection. This may be particularly important in the NT setting where HBV sub-genotype C4, the only sub-genotype documented in Aboriginal people, has a serotype mismatch with the HBV vaccine [12]. Emerging evidence suggests that despite the existing subtype mismatch, the vaccine is largely effective in preventing against chronic infection but it is sub-optimal at protecting against Anti-HBc positivity [42] It is still unclear if this is clinically important [42].

HBV testing on the post vaccine cohort would have significant workload implications as, to date only 600 people (20.4%) have had a HBV test, with four people HBsAg positive (0.7%). This proportion of HBsAg positive HBV tests may be higher than the true prevalence of CHB in the whole of the post vaccination population as testing is usually risk-based in this cohort. We would predict that the prevalence in the post vaccine cohort is more likely to be closer to the 0.2% found in this study. We are less certain about the Anti-HBc positivity given vaccine mismatch [11, 12]. Limited serological testing of the population born after 1990 constrains the ability to address uncertainty resulting from mismatch between the dominant HBV C4 sub-genotype and available vaccines. Ongoing work, generated by this study, will be looking at serology in this cohort in a higher prevalence region.

The results found in the five selected communities may not be representative of all Top End communities. The communities selected for the quality assurance step of this study were already engaged in the HBV coding process and three of the authors worked in these communities. For these reasons, it is likely that the HBV testing, vaccinations and pre-existing HBV status documentations may be over-representative. We believe there is likely regional and community variation in testing and that our study population may offer an over-estimate of the testing and HBV status allocation that is occurring in other parts of the NT.

The level of missing HBV test and vaccination data on the linked dataset from the EHR patient databases raises concerns about the accuracy of interpreting an individual’s HBV status. It also highlights why the data linkage and computerised algorithmic coding process cannot be applied to direct clinical care without manual review in the study context. However, if systematic processes are established prospectively to allow for capture and extraction of this data, there would be enormous potential to use data linkage and our computerised algorithm for direct clinical care in the future. Unfortunately, large retrospective datasets are not fit for this specific purpose. There is currently a new whole-of-health EHR systems being developed in the NT. Having been through this data linking and manual review process we are now working with the developers to provide advice and direction to improve the likelihood that the new EHR system will have complete and accurate data extractability and transparency that would provide the utility to apply this computerised algorithm moving forward. If there are established vaccination programs in place, such as in the NT, the determination of an individual’s HBV status is mostly a one-off process. Therefore, prospective maintenance would be minimal and would only need to occur for new people coming into the NT, including new babies.

The process of a clinician manually reviewing all available data used as the quality assurance tool, such as in this study, is highly accurate but labour intensive. However, we determined that this manual review process will be used in the same way for the remaining Aboriginal population of the NT. In total, we will review a further approximately 54,000 people to ensure no case of CHB is left unknown and to improve the cascade of care, health and well-being of Aboriginal people living with CHB in the NT.

## Conclusion

Despite high agreement (99.9%) in accurately linking individuals from various databases, the level of missing data from pathology and vaccination datasets raises concerns about the completeness of the data. The error rate detected (17.7%) on the HBV code generated by the computerised algorithm in the linked dataset was considered too high to be applied for direct clinical care. Through the process of a clinician manually reviewing all data sources, we have a clearer understanding of the CHB prevalence and the HBV status of the population in these communities.

## Supporting information

S1 Appendix Decision tree for the algorithm to automate and assist with clinical decisions using vaccination and pathology data. (PDF)

S2 Appendix Description of the algorithm development process and iterations. (PDF)
