# Supplementary material for: Data linkage and computerised algorithmic coding to enhance individual clinical care for Aboriginal people living with chronic hepatitis B in the Northern Territory of Australia – Is it feasible?
Source: PLoS One. 2020 Apr 28;15(4):e0232207. doi: 10.1371/journal.pone.0232207 (PMC7188233; doi:10.1371/journal.pone.0232207)
Supplement: S1 Appendix — (PDF) [file pone.0232207.s001.pdf]

S1 Appendix. Decision tree for the algorithm to automate and assist with clinical decisions using vaccination and pathology data

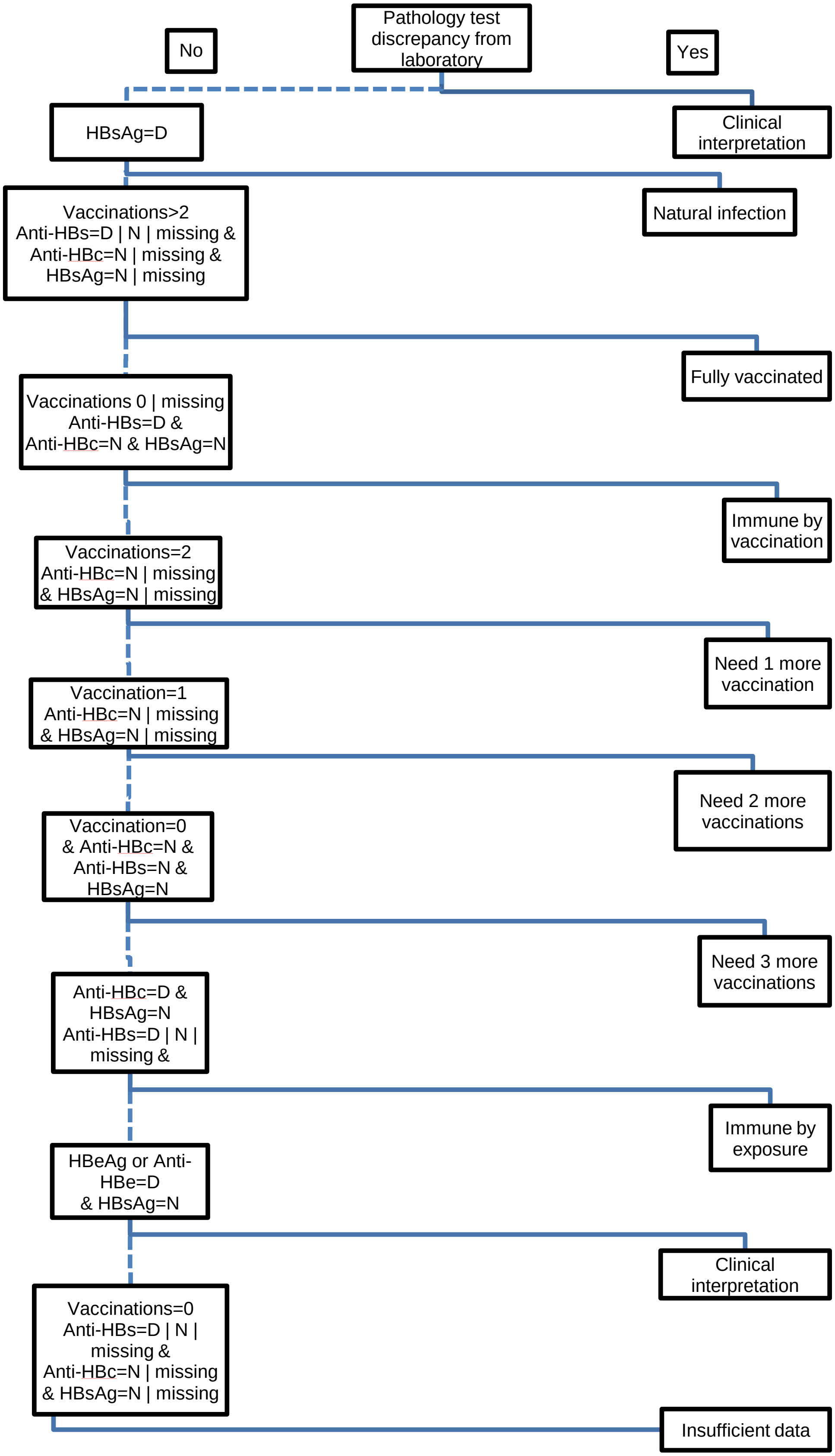

S1 Appendix. Decision tree for the algorithm to automate and assist with clinical decisions using vaccination and pathology data

Legend:

D = detected / positive

N = not detected / negative

| = or

HBsAg = hepatitis B surface antigen, if positive indicates active infection

Anti-HBc = hepatitis B core antibody, if positive is a marker of immunity from infection (past or current)

Anti-HBsAb – hepatitis B surface antibody, if positive (>10IU/ml) indicates immunity (from vaccination or infection)

HBeAg & Anti-HBe = hepatitis B “e” antigen/antibody if positive are markers of infection
