# Supplementary material for: Data linkage and computerised algorithmic coding to enhance individual clinical care for Aboriginal people living with chronic hepatitis B in the Northern Territory of Australia – Is it feasible?
Source: PLoS One. 2020 Apr 28;15(4):e0232207. doi: 10.1371/journal.pone.0232207 (PMC7188233; doi:10.1371/journal.pone.0232207)
Supplement: S2 Appendix — (PDF) [file pone.0232207.s002.pdf]

Supplementary information

S2 Appendix. Description of the algorithm development process and iterations

Development of coding algorithm

Initial development of the coding algorithm by 2 authors (GS and JYS) was conducted from August 2015 using STATA v13. Trial algorithms were tested using data from a single remote community, later expanded to other communities within the same region. A similar methodology was followed:

1. A computerised algorithm was used to allocate a HBV code to each individual
2. Quality assurance was then conducted using manual interrogation of individual EHRs by a trained RAN with oversight of the project medical officer (GS)
3. Sources of error were identified and used to refine the algorithm further
4. Steps 1-3 were then repeated

Table 1: The initial algorithm contained 8 codes, August 2015

	Code no.	Vaccs	HBsAg	Anti-HBc	Anti-HBs
Hep B; Non-immune	1	No	Neg	Neg	Neg
Hep B; Fully vaccinated	2	Yes x3 doses regardless of interval/dose	Neg	Ignore	Neg or pos
Hep B; partially vaccinated, needs 1 dose	3	x2 doses of regardless of interval/dose	Neg	Neg	Neg
Hep B; partially vaccinated, needs 2 doses	4	x1 dose of regardless of interval/dose	Neg	Neg	Neg
Hep B; Immune by Exposure	5	No	Neg	**Pos**	No
Hep B; Infected	6	Ignore	**Pos**	Pos	Neg or pos
Clinician interpretation req'd	7	Yes- any number	**Neg**	Pos	Neg or pos
Undetermined- needs serology	8	No	-	-	-

Notes:
If HBsAg pos apply code 6 regardless of other data

If Anti-HBs pos, Anti-HBc neg and NO vaccinations apply code 2
This process identified error and omission in the source dataset, some of which were able to be corrected for subsequent iterations.
Several coding discrepancies were identified:
Anti-HBc+ in presence of vaccinations
Anti-HBs+ in absence of vaccination
the issue of Anti-HBc results varying over time and which result is used to determine the serocode.

As a result, the number of computer-generated codes expanded to address coding discrepancies identified during the QA process.

Table 2: Second iteration of algorithm, August 2016

	Code no.	Vaccs	HBsAg	Anti-HBc	Anti-HBs
Non-immune	1	None or missing	Missing or neg	Neg	Neg
Fully vaccinated	2	x3 doses regardless of interval/dose	Missing or Neg	Missing or Neg	ignore
Partially vaccinated, needs 1 dose	3	x2 doses of regardless of interval/dose	Missing or neg	Missing or neg	Missing or neg
Partially vaccinated, needs 2 doses	4	x1 dose of regardless of interval/dose	Missing or neg	Missing or neg	Missing or neg
Immune by Exposure	5	ignore	Neg	**Pos**	Any result
Chronic infection	6	Ignore	**Pos**	Any result	Neg or pos
Discrepant result: needs interpretation	7	Yes- any number	**Neg**	Pos	Neg or pos
No data	8	missing	missing	missing	missing
Insufficient data- needs serology	9	No	-	-	-
Immune by imm: presumed fully immunised	10	Missing or < 3	Missing or neg	Missing or neg	Pos (ie >10)
Discrepant core antibody	11				

Notes:

If HBsAg pos apply code 6 regardless of other data.

HBcAb, Code 11

There are individuals with discordant Anti-HBc results (earlier positive test results turning negative in later tests)

Code 10

There are individuals for whom there is a positive surface antibody result and less than 3 immunisations recorded or for whom the only data available is Anti-HBs pos.

Code 7

These are people with discrepant results: i.e. ones that do not make sense. A clinician will need to review and order new tests to sort them out on a case by case basis.

- Pos for all 3 of Anti-HBc, HBsAg and Anti-HBs
- HB e antigen pos but negative for HBsAg
- HB e antibody pos but negative for all 3 of Anti-HBc, HBsAg and Anti-HBs
- HBsAg pos and Anti-HBc neg
- HBsAg pos and Anti-HBs pos

Code 8

This is a left-over code from all the others. These people have results but not enough to be able to classify them into one of the other categories. On a case by case basis, extra tests will need to be ordered for them to get classified.

Coding Algorithm order

Each new line excludes records that have already been assigned a code

Therefore, for example as all HBsAg pos people are coded as "6" in the first instance, they cannot be recoded as anything else so all other codes will not contain anyone who is HBsAg pos

The exception to this is code 7 which contains discrepant results.

HBsAg pos→Code 6

>=3 Vac → Code 2

Anti-HBs pos, and < 3 vaccines and not HBcAb pos or missing and HBsAg not pos or missing→ code 10

>=3 Vac and not HBcAb pos → Code 2

2 Vac and not HBcAb pos→ Code 3

1 Vac and not HBcAb pos→ Code 4

No Vac and Anti-HBs neg and Anti-HBc neg →Code 1

Anti-HBc pos→Code 5

Specific discrepant combinations (see above) and not excluding records not already coded →Code 7

Code remaining missing→Code 8

Table 3: Timeline during QA process

Date:	Action and finding	Change / outcome
16/08/2016	Algorithm (Table 2) and dataset (n=19,317) sent to Study Nurse (MM) for initial Quality Assurance (QA)	
20/08/2016	Subset of data 5 pilot communities (6,728). 200 patients randomly selected from subset for QA	
09/09/2016	Error detected by clinician:<br>For Code 1 HBsAg should be only negative (not missing) data. If HBsAg missing the person should be allocated to code 8 – insufficient data.	This error was rectified in algorithm and 450 people (out of 19283) went from "non-immune" to "insufficient data"
23/09/2016	33/199 (16.6%) incorrect code of which 29 (87.9% of errors) were related to failing to pick up / incorrect vaccination records.<br><br>15 - Failed to pick up primary care EHR<br><br>3 - Failed to pick up NT Immunisation register record<br><br>11 - Unclear which: both primary care EHR and NT Immunisation register have same number of immunisations recorded but this not correctly coded<br><br>Support to study nurse and QA on study nurse's interpretations of se HBV status by senior clinicians (SS, KH) undertaken – 100% accuracy detected on 30 randomly selected people.	Requested primary care EHR to extract vaccination data again
02/10/2016	A further randomly selected 200 people chosen on newly fixed dataset.QA done	
02/12/2016	34/200 (17%) incorrect coding on next 200.<br><br>Noted that people were being allocated to Code 2 fully vaccinated even if vaccination doses given too closely together (and therefore invalid dose). If 3 valid doses not received person should be allocated to code 8 "insufficient data", see table 4	Clarified the vaccine dosage minimum intervals and applied these to the algorithm.
20/01/2017	Adjusted dataset (with new algorithm applied) returned to study nurse to complete QA on the remaining subset of data	Completed QA as reported in Fig 2 and Table 4 of manuscript

Table 4: The final algorithm tested incorporated 2 additional codes and changes made as a result of QA by study nurse, Dec 2016

Code number	HBV status	Vaccinations	HBsAg[a]	Anti-HBc[b]	Anti-HBs[c]
**1**	Non-immune	None or missing	Negative	Negative	Negative
**2**	Fully Vaccinated	3 doses	Missing or negative	Missing or negative	Missing or negative
**3**	Partially vaccinated, needs 1 dose	2 doses	Missing or negative	Missing or negative	Missing or negative
**4**	Partially vaccinated, needs 2 doses	1 dose	Missing or negative	Missing or negative	Missing or negative
**5**	Immune by exposure	Ignore	Negative	**Positive**	Negative, positive or missing
**6**	Chronic infection	Ignore	**Positive**	Negative, positive or missing	Negative, positive or missing
**7**	Clinical interpretation required[d]	Yes, any number	Negative or Positive	**Positive** or Negative	Negative or positive
**8**	No data	None or missing	Missing	Missing	Missing
**9**	Insufficient data	None or missing	Missing or negative	Missing or negative	Missing or negative
**10**	Presumed fully immunised	None or missing	Negative	Negative	Positive (i.e. >10)
**11**	Discrepant core antibody	Needs interpretation			

[a] HBsAg – hepatitis B surface antigen, if positive indicates active infection, [b] Anti-HBc – hepatitis B core antibody, if positive is a marker of immunity from infection (past or current), [c] Anti-HBsAb – hepatitis B surface antibody, if positive (>10IU/ml) indicates immunity (from vaccination or infection), [d] Individuals with discrepant results that need a clinician to review in order to interpret, including scenario where hepatitis B "e" antigen/antibody is positive (markers of infection) and HBsAg negative

Further refinements to the algorithm were made after the study was complete, when analysing all sources of potential error. Several limitations in the computerised algorithm were identified throughout the various iterations of both algorithm and clinical manual review audit process. In line with contemporary NT hepatitis B public health and vaccination guidelines the algorithm allocated 3 HBV containing vaccinations to the "fully vaccinated" code. We identified that such an approach failed to consider appropriate dose intervals for vaccination and that 62 individuals received vaccinations at intervals that were less that the minimum dosing intervals and were thus invalid (this had been incorporated into the

version in Table 5). Additionally, the algorithm failed to distinguish individuals vaccinated according to the birth and infancy vaccination schedule and children and adults vaccinated later in life. These indiviuals require serology testing to ensure they have not become HBV infected during the period in which they were susceptible to HBV prior to vaccination. Given the natural history of CHB, that acquisition in infancy and childhood is more likely to lead to chronical infection, there would potentially be CHB infections in this cohort. In the manual review process this potential was considered (and adjusted for when calculating the error rate per code).

On further review and discussion with the designer of the algorithm, practically code 7 and code 11 could be amalgamated, reflected in refined algorithm in Table 5.

Table 5: The final iteration of algorithm, refinements made after reviewing n=5,293, analysing sources of error and in discussion with researchers of this study 2019-20

**Code number**	**HBV status**	**Vaccinations**	**HBsAg**[a]	**Anti-HBc**[b]	**Anti-HBs**[c]
**1**	Non-immune	None or missing	Negative	Negative	Negative
**2**	Fully Vaccinated	3 doses	Missing or negative	Missing or negative	Negative, positive or missing
**3**	Partially vaccinated, needs 1 dose	2 doses	Missing or negative	Missing or negative	Negative, positive or missing
**4**	Partially vaccinated, needs 2 doses	1 dose	Missing or negative	Missing or negative	Negative, positive or missing
**5**	Immune by exposure	Prioritise serological markers	Negative	**Positive**	Negative, positive or missing
**6**	Chronic infection	Prioritise serological markers	**Positive**	Negative, positive or missing	Negative, positive or missing
**7**	Clinical interpretation required[d]	Prioritise serological markers	Negative or Positive	**Positive** or Negative	Negative or positive
**8**	No data	None or missing	Missing	Missing	Missing
**9**	Insufficient data	None or missing	Missing or negative	Missing or negative	Missing or negative
**10**	Presumed fully immunised	None or missing	Negative	Negative	Positive (i.e. >10)

[a] HBsAg – hepatitis B surface antigen, if positive indicates active infection
[b] Anti-HBc – hepatitis B core antibody, if positive is a marker of immunity from infection (past or current)
[c] Anti-HBs – hepatitis B surface antibody, if positive (>10IU/ml) indicates immunity (from vaccination or infection)
[d] Individuals with discrepant results that need a clinician to review in order to interpret, including previous Anti-HBc positive but most recent test result Anti-HBc neg

Emphasis was placed on the requirement for clinical utility and accuracy of the coding was validated using the QA methodology due to implications in patient care (see Table 6).

Table 6. Automated Status HBV coding and corresponding clinical pathway and preferred code.

Automated Coding	Clinical pathway and serostatus coding
Hepatitis B; infected	Code added:<br>Hepatitis B; infected; NOT on treatment if tenofovir or entecavir NOT prescribed<br>Hepatitis B; infected; on treatment if tenofovir or entecavir prescribed.<br>Summary list sent to senior clinician<br>PCIS records reviewed and recall added to record for MO review where needed.
Hepatitis B; immune by exposure	Code added:<br>Hepatitis B; immune by exposure<br>No further action
Clinical Interpretation required	PCIS record interrogated by project MO to determine appropriate code. This included examining records regarding the timing of the Anti-HBc (+) result in relation to the timing of vaccination and checking mother's record to check for HBsAg (+)
Hepatitis B; fully vaccinated	Code added:<br>Hepatitis B; fully vaccinated<br>No further action
Hepatitis B; partially vaccinated, needs 1 dose	Code added:<br>Hepatitis B; non-immune<br>Recall added for additional dose
Hepatitis B; partially vaccinated, needs 2 doses	Code added:<br>Hepatitis B; non-immune<br>Recall added for additional doses
Hepatitis B; non-immune	Code added:<br>Hepatitis B; non-immune<br>PCIS HBV vaccination care plan added
Hepatitis B; undetermined	Code added:<br>Nil<br>List of patients provided to PHC staff to conduct recall for testing

Establishment of the database occurred in September 2016 using primary clinical data sources as outlined in Fig 1 of the manuscript. Custodianship of the database is maintained

by Top End Health Service. Ongoing work of the HBV Status Project has established a continuous quality improvement (CQI) mechanism to periodically report on HBV statuses and support primary care clinicians.
